# An Asymptomatic SARS-CoV-2-Infected Infant With Persistent Fecal Viral RNA Shedding in a Family Cluster: A Rare Case Report

**DOI:** 10.3389/fmed.2020.562875

**Published:** 2020-09-25

**Authors:** Shen Chen, Jiafeng Si, Wenqiang Tang, Anqi Zhang, Li Pan, Meng An, Huawei Zhang, Shoukun Xue, Kunpeng Wu, Shuangfeng Chen, Wei Zhang, Wei Liu, Bo Fu

**Affiliations:** ^1^Department of Breast and Thyroid Surgery, Liaocheng People's Hospital, Liaocheng, China; ^2^Department of Clinical Laboratory, Dong'e People's Hospital, Liaocheng, China; ^3^Department of Central Laboratory, Liaocheng People's Hospital, Liaocheng, China; ^4^Department of Clinical Laboratory, Liaocheng People's Hospital, Liaocheng, China; ^5^Department of Thoracic Breast and Thyroid Surgery, Liaocheng Infectious Disease Hospital, Liaocheng, China; ^6^Department of Breast and Thyroid Surgery, Shandong Maternal and Child Health Hospital, Jinan, China; ^7^Department of CT, Liaocheng People's Hospital, Liaocheng, China

**Keywords:** SARS-CoV-2, COVID-19, asymptomatic, fecal viral RNA shedding, family cluster

## Abstract

An outbreak of coronavirus disease 2019 (COVID-19), caused by severe acute respiratory syndrome coronavirus 2 (SARS-CoV-2), has become pandemic worldwide. A better understanding of asymptomatic infections is crucial to prevent and control this epidemic. Here, we report the epidemiological and clinical characteristics of a family cluster with SARS-CoV-2 infection. In the family cluster, a 32-year-old male (case 1) and a 53-year-old female (case 2, the mother-in-law of case 1) exhibited clinical symptoms of COVID-19, while case 1's 32-year-old wife (case 3) and their 11-month-old daughter (case 4) were both asymptomatic. Notably, case 4's nasopharyngeal swab samples was negative for nearly 80 days, and her immune system has been boosted for at least 57 days, but the fecal samples have tested positive for 100 days (May 13, 2020), suggesting SARS-CoV-2 may invade enterocytes and may exist in individuals with low antiviral immunity for a long term. This report highlights that asymptomatic infections should be managed with caution and vigilance. For SARS-CoV-2 testing of asymptomatic cases, besides the normally used nasopharyngeal swab, fecal sample testing is also needed.

## Introduction

An outbreak of coronavirus disease 2019 (COVID-19), caused by severe acute respiratory syndrome coronavirus 2 (SARS-CoV-2), has become pandemic worldwide. Although most individuals with SARS-CoV-2 infection exhibit clinical symptoms, a few [1.2% in China (1)] cases do not. Mounting evidence indicates asymptomatic carriers may still be infectious (2–4). Given that asymptomatic cases are not easily identified from the population, they are often neglected by infection prevention and control protocols, and they therefore have a greater potential transmission risk. A better understanding of asymptomatic infections is crucial to prevent and control of this epidemic. Here, we report the epidemiological and clinical characteristics of a family cluster with SARS-CoV-2 infection, notably including an 11-month-old infant with persistent fecal viral RNA shedding for 100 days until now (May 13, 2020).

## Case Presentation

**Figure 1:**
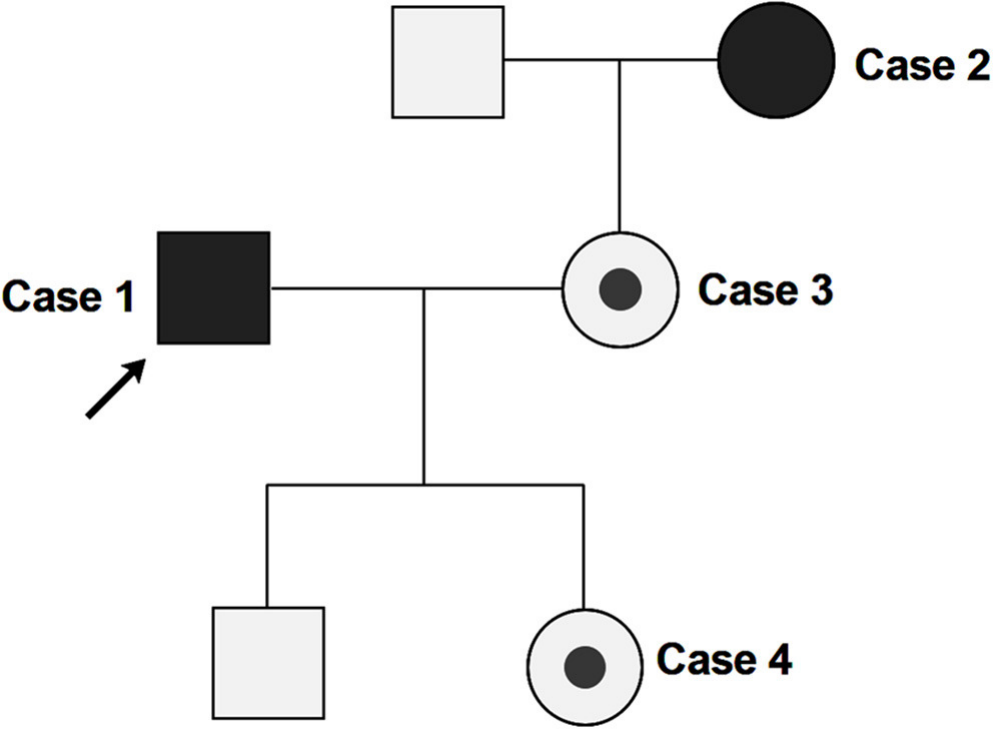
Pedigree of the clustered SARS-Cov-2 infection cases. Arrow indicates the proband (case 1). Case 2, 3, and 4 are close contacts of case 1. Full black fills indicate the individuals with COVID-19 symptoms (case 1 and 2), while partial black fills indicate asymptomatic carriers of SARS-CoV-2 (case 3 and 4).

In this family cluster, a 32-year-old male (case 1) and a 53-year-old female (case 2, the mother-in-law of case 1) exhibited clinical symptoms, while case 1's 32-year-old wife (case 3) and their 11-month-old daughter (case 4) were both asymptomatic (Figure 1).

**Figure 2:**
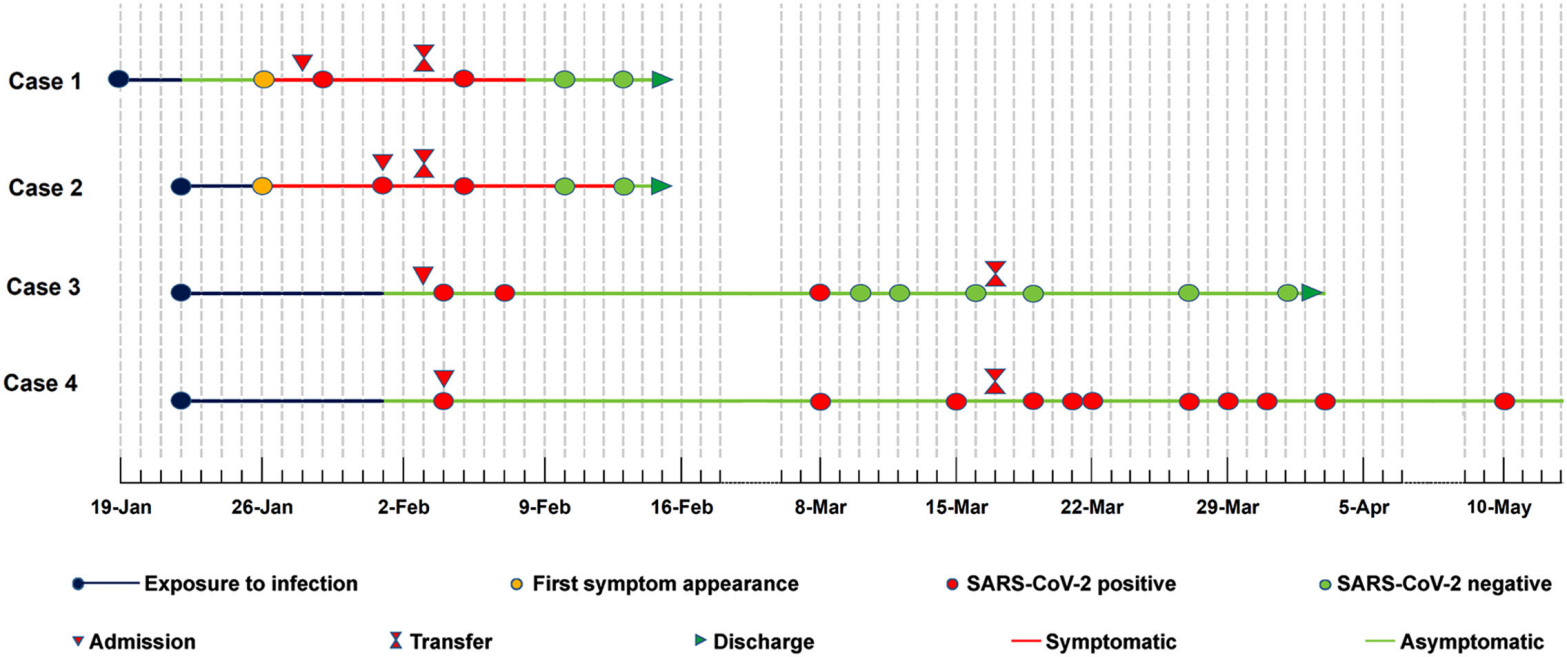
Timeline of the cases exposed to SARS-CoV-2.

**Figure 3:**
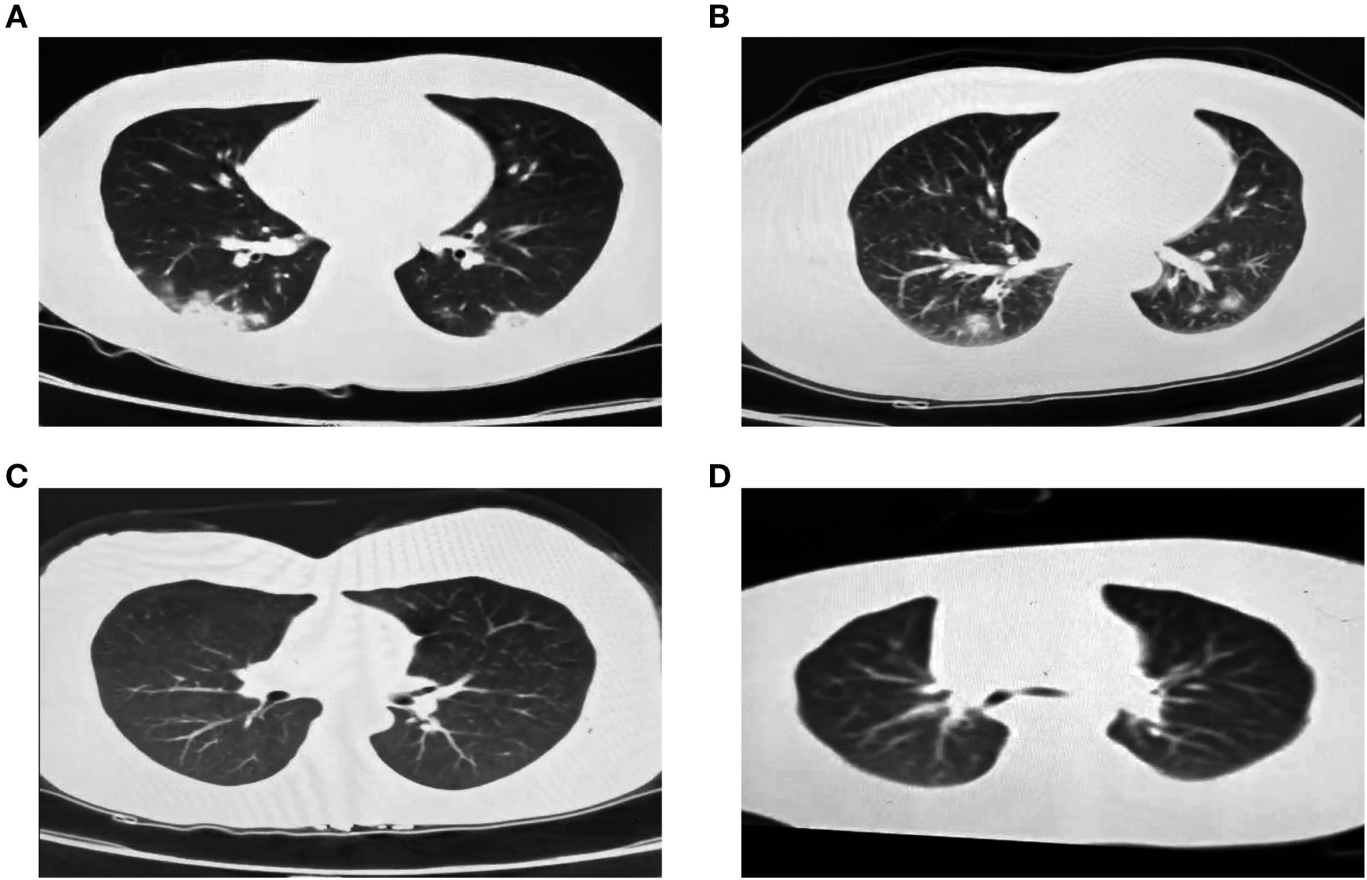
Chest CT images of the four family-clustered cases. **(A)** Case 1. **(B)** Case 2. **(C)** Case 3. **(D)** Case 4.

On Jan 19, 2020, case 1 (index patient) visited Wuhan (Hubei, China) for a meeting and then took a train from Wuhan on Jan 21 and arrived at Liaocheng (Shandong, China) on Jan 22 (Figure 2). He had a fever of 37.8°C and a productive cough after 4 days (Figure 2 and Table 1). On Jan 28, he was taken to Dong'e People's Hospital (Liaocheng, Shandong, China) by ambulance. After pre-screening and triage, he was admitted to the Infectious Disease Unit for isolation as a suspected case. His chest computed tomogram (CT) scans showed multiple mottling and ground-glass opacities in the bilateral lung (Figure 3A), suggesting this patient has developed to pneumonia. SARS-CoV-2 nucleic acid tests (ORF1ab and N genes) of the nasopharyngeal swab samples were positive by qRT-PCR (BioGerm, Shanghai, China). According to the instruction, the kit covers 100% of known COVID-19 sequences and has no cross-reactivity with other pathogens, and the limit of detection of the kit is 1,000 copies/mL. His clinical classification was moderate. Remission was achieved after treatment with antiviral and traditional Chinese medicine.

**Table 1:** Epidemiological and clinical characteristics of the family-clustered cases with SARS-CoV-2 infection.

**Characteristics**	**Case 1**	**Case 2**	**Case 3**	**Case 4**
Age	32 years	53 years	32 years	11 months
Gender	Male	Female	Female	Female
**Symptoms at admission**				
Fever	√	√	×	×
Chills	×	×	×	×
Cough	√	√	×	×
Sore throat	×	×	×	×
Rhinorrhea	×	×	×	×
Diarrhea	×	×	×	×
**Chest CT scan**				
Mottling and ground-glass opacity	√	√	×	×
**Blood analysis**				
Leukocytes (×10^9^/L; normal range 5–12)	4.85	6.46	7.00	11.35
Neutrophils (×10^9^/L; normal range 1.8–6.3)	3.07	4.74	4.56	1.33
Lymphocytes (×10^9^/L; normal range 1.1–3.2)	1.37	1.30	1.88	9.27
CRP (mg/L; normal range 0–10)	10.90	22.70	0.50	3.00
**Antibody of respiratory pathogen test**				
Influenza A	×	×	×	×
Influenza B	×	×	×	×
Parainfluenza	×	×	×	×
Respiratory syncytial virus	×	×	×	×
Adenovirus	×	×	×	×
Mycoplasma pneumoniae	×	×	×	×
Chlamydia pneumoniae	×	×	×	×
Legionella pneumophila	×	×	×	×

*√, with; ×, without; CRP, C-reactive protein*.

Case 2 was a close contact of case 1 from Jan 22 to 26, and afterwards she developed to a fever of 38.9°C and cough (Figure 2). On Feb 1, she visited the hospital for diagnosis and treatment. Her chest CT showed multiple inflammatory changes and bronchitis in the bilateral lung (Figure 3B). Her nasopharyngeal swab sample was detected with positive SARS-CoV-2 RNA. She was classified as a moderate case. After antiviral treatment and supplement of albumin and human immunoglobulin, her symptoms were gradually relieved. After two consecutive negative SARS-CoV-2 nucleic acid tests of nasopharyngeal swabs with 24 h interval, case 1 and 2 were discharged on Feb 15 (Figure 2).

As close contacts of case 1 and 2, case 3, and 4 visited the Fever Clinic for further examination on Feb 4 (Figure 2). Both individuals did not show any clinical symptoms, such as fever, chills, cough, sore throat, rhinorrhea, or diarrhea, and no signs of pneumonia was observed on chest CT scans (Table 1 and Figures 3C,D). However, their nasopharyngeal swab and fecal samples were both tested positive for SARS-CoV-2. On the same day, the two cases were admitted to the Infectious Disease Unit for isolation as asymptomatic cases. After 36 days of isolation and observation, case 3's nasopharyngeal swab and fecal samples turned negative for SARS-CoV-2 (Figure 2).

For case 4, her nasopharyngeal swab samples turned negative after 22 days of isolation, but the fecal samples have tested positive until now (100 days by May 13; Figure 2). Repeated blood routine examination showed decreased neutrophils and elevated lymphocytes (Supplementary Table 1), indicating a persistent viral infection. Flow cytometry showed that the counts of NK cells, B cells, and T-cell subtypes were all out of the reference ranges (Supplementary Table 1), indicating activated innate and adaptive immunity responses. The SARS-CoV-2 specific IgG was detectable on Mar 18 and Apr 12, indicating an active humoral immune reaction against the virus (5, 6).

## Discussion

The transmission concealment, symptom subjectivity and detection limitations increase the transmission risk of asymptomatic infections. In this report, case 2 and case 1 appeared symptoms of infection almost at the same time, suggesting that the infection seems to be transmitted during the incubation period of the index patient. To the best of our knowledge, case 4 represents the longest SARS-CoV-2 virus RNA shedding (100 days) in asymptomatic carriers. Although RT-PCR-based SARS-CoV-2 tests of the nasopharyngeal swab samples have turned negative for nearly 80 days, and the infant's immune system has been boosted for nearly 60 days, her fecal samples have still been SARS-CoV-2 positive.

Currently, case 4 has no respiratory symptoms; considering the negative SARS-CoV-2 of nasopharyngeal swab samples, the viruses might have been eliminated from the respiratory system. Since RNA fragments' half-life is very short (7), we speculate that SARS-CoV-2 viruses, rather than the RNA fragments, still exist, which is supported by the reduced neutrophils and elevated lymphocytes. As fecal samples have been constantly positive, we suspect that the viruses may be present in the gastrointestinal tract. Previous studies have shown that angiotensin-converting enzyme 2 (ACE2), a critical SARS-CoV-2 receptor (8), is not only expressed in the lung cells but also in the enterocytes (9).

Even though the enterocytes of case 4 may have viruses, she did not show any gastrointestinal symptoms, such as diarrhea, abdominal pain, nausea, or vomiting. Despite the production of IgG, the immune function of infants under 3-year-old has not been fully developed (10), which could be the reason for incomplete elimination of SARS-CoV-2 viruses in this case. Although carrying viruses, the body might be in an immune-tolerance stage and as such may not present related symptoms. It is still unpredictable whether and when the SARS-CoV-2 infection would turn negative in this case. It is still unknown whether SARS-CoV-2, like hepatitis B, could exist in individuals with low anti-viral immunity for a long time and who may become long-term carriers.

The infectivity, transmissibility, and epidemiology of asymptomatic carriers have not been clearly illustrated, and further knowledge is urgently needed. Nevertheless, before thorough understanding, asymptomatic infections still should be managed with caution and vigilance. According to the clinical experience management for asymptomatic carriers, here are some suggestions: (1) pay close attention to asymptomatic infections with intensive monitor and detection; (2) similar to the confirmed COVID-19 patients, asymptomatic carriers should also be isolated and observed until the nucleic acid test turns negative; (3) close contacts of asymptomatic carriers should also be screened for SARS-CoV-2 infection; and (4) for the SARS-CoV-2 nucleic acid test, besides the normally used nasopharyngeal swab, fecal sample-testing is also needed.

## Data Availability Statement

All datasets generated for this study are included in the article/Supplementary Material.

## Ethics Statement

Written informed consent was obtained from the individual(s), and minor(s)' legal guardian/next of kin, for the publication of any potentially identifiable images or data included in this article.

## Author Contributions

SheC, JS, WT, LP, MA, HZ, SX, and KW collected the samples and contributed to data acquisition. BF, ShuC, WZ, and WL designed the study. SheC, AZ, and BF wrote and edited the paper. All authors read and approved the final manuscript.

## Conflict of Interest

The authors declare that the research was conducted in the absence of any commercial or financial relationships that could be construed as a potential conflict of interest.

## Abbreviations

COVID-19: coronavirus disease 2019
SARS-CoV-2: severe acute respiratory syndrome coronavirus 2
CT: computed tomogram
ACE2: angiotensin-converting enzyme 2.
