# Supplementary material for: An Asymptomatic SARS-CoV-2-Infected Infant With Persistent Fecal Viral RNA Shedding in a Family Cluster: A Rare Case Report
Source: Front Med (Lausanne). 2020 Sep 25;7:562875. doi: 10.3389/fmed.2020.562875 (PMC7546332; doi:10.3389/fmed.2020.562875)
Supplement: Supplementary file 1 [file Table_1.DOCX]

**Supplementary Tables**

**Supplementary Table S1** Blood analysis results of case 4 at various time points.

| **Characteristics** | **5-Feb^a^** | **5-Mar^a^** | **10-Mar^a^** | **27-Mar^b^** | **2-Apr^b^** | **12-Apr^b^** |
| --- | --- | --- | --- | --- | --- | --- |
| Leukocyte (× 10^9^/L) | 11.35 | 9.65 | 10.31 | 12.18 | 10.41 | 10.35 |
| Neutrophil (× 10^9^/L) | 1.33 | 1.50 | 1.55 | 4.88 | 2.44 | 3.16 |
| Neutrophil ratio (%) | 11.60 | 15.60 | 15.00 | 40.09 | 23.41 | 30.49 |
| Lymphocyte (× 10^9^/L) | 9.27 | 7.59 | 8.18 | 6.32 | 7.20 | 6.43 |
| Lymphocyte ratio (%) | 81.70 | 78.70 | 79.30 | 51.89 | 69.24 | 62.12 |
| CRP (mg/L) | 3.00 | 0.20 | 0.20 | 2.21 | 1.39 | 0.50 |
| NK cell (× 10^3^/mL) | NA | NA | NA | 1294 | NA | NA |
| B cell (× 10^3^/mL) | NA | NA | NA | 1626 | NA | NA |
| CD3+T cell (× 10^3^/mL) | NA | NA | NA | 6249 | NA | NA |
| CD4+T cell (× 10^3^/mL) | NA | NA | NA | 4663 | NA | NA |
| CD8+T cell (× 10^3^/mL) | NA | NA | NA | 1347 | NA | NA |

^a^ normal range: leukocyte: 5-12; neutrophil: 1.8-6.3; neutrophil ratio: 40-70; lymphocyte: 1.1-3.2; lymphocyte ratio: 20-50; CRP: 0-10. ^b^ normal range: leukocyte: 4-10; neutrophil: 2.0-7.7; neutrophil ratio: 45-77; lymphocyte: 0.8-4.0; lymphocyte ratio: 20-40; CRP: 0.5-10; NK cell: 175-525; B cell:180-324; CD3+T cell: 1185-1901; CD4+T cell: 561-1137; CD8+T cell: 404-754. CRP, C-reactive protein. NA, not available.
